# B-Doped g-C_3_N_4_/Black TiO_2_ Z-Scheme Nanocomposites for Enhanced Visible-Light-Driven Photocatalytic Performance

**DOI:** 10.3390/nano13030518

**Published:** 2023-01-28

**Authors:** Yuwei Wang, Kelin Xu, Liquan Fan, Yongwang Jiang, Ying Yue, Hongge Jia

**Affiliations:** Heilongjiang Provincial Key Laboratory of Polymeric Composite Materials, College of Materials Science and Engineering, Qiqihar University, Qiqihar 161006, China

**Keywords:** photocatalysis, black TiO_2_, B-doped g-C_3_N_4_, Z-scheme heterojunction, oxygen vacancy defect

## Abstract

Black TiO_2_ with abundant oxygen vacancies (OVs)/B-doped graphitic carbon nitride (g-C_3_N_4_) Z-scheme heterojunction nanocomposites are successfully prepared by the one-pot strategy. The OVs can improve not only photogenerated carrier separation, but also the sorption and activation of antibiotic compounds (tetracycline hydrochloride, TC). The prepared heterojunction photocatalysts with a narrow bandgap of ∼2.13 eV exhibit excellent photocatalytic activity for the degradation of tetracycline hydrochloride (65%) under visible light irradiation within 30 min, which is several times higher than that of the pristine one. The outstanding photocatalytic property can be ascribed to abundant OVs and B element-dope reducing the bandgap and extending the photo-response to the visible light region, the Z-scheme formation of heterojunctions preventing the recombination of photogenerated electrons and holes, and promoting their effective separation.

## 1. Introduction

In recent years, the main aspects of environmental problems have been the energy crisis and pollution, with water pollution receiving special attention [1,2,3]. Water pollution is principally caused by heavy-metal ion contaminants and organic pollutants such as hormones, dyes, aromatics, pesticides, and perfluorinated organic compounds (PFOCs). Organic pollutants in wastewater, for example, have high toxicity, carcinogenicity, and refractory degradation, posing a significant threat to human health. As a consequence, it is critical to develop efficient technologies for breaking down organic pollutants from water [4]. Photocatalysis, one of the advanced oxidation methods for producing highly oxidizing free radicals, has been identified as a sustainable and ecologically friendly method for the degradation of pollutants. Photocatalytic oxidation has been acknowledged as a significant and successful candidate for eliminating poisonous and harmful contaminants in aqueous environments [5,6,7,8,9].

Numerous photocatalytic materials with superior band structures, visible light adsorption, charge separation, and transport have been created to date. Due to its huge band gap, the original TiO_2_—being a mature semiconductor—can only be used to purify wastewater using ultraviolet light, regardless of the fact that TiO_2_ has much lower biotoxicity than the majority of semiconductors [6,10,11,12]. Fortunately, Chen et al. discovered black TiO_2_ nanomaterials through a surface hydrogenation strategy, which narrowed the bandgap and extended photo-absorption from ultraviolet to visible light and/or near-infrared [13,14,15,16]. The outstanding solar-driven photocatalytic performance represented a breakthrough for wide-spectrum response TiO_2_ materials. In recent years, graphitic carbon nitride (g-C_3_N_4_), with a narrow band gap, excellent stability, and fast charge transfer, has been considered a potential visible light photocatalyst since the groundbreaking work reported by Wang et al. in 2009 [17,18,19,20]. However, the quick electron-hole recombination, low quantum efficiency, insufficient specific surface area, and other issues continue to restrict the photocatalytic activity of g-C_3_N_4_ [21]. Recently, many groups have documented the use of P- or S-doped g-C_3_N_4_ to enhance photocatalytic activity [22,23,24]. Wang et al. also discovered that even a small amount of boron doping could significantly increase photocatalytic activity [25].

Here, we proposed a one-pot synthesis of a B-doped g-C_3_N_4_/black-TiO_2_ (BCBT) heterojunction nanocomposite photocatalyst using NaBH_4_ as a solid reducing agent. This catalyst showed significantly higher photocatalytic degradation activity of high-toxic tetracycline hydrochloride (TC) when exposed to both visible light and simulated sunlight. The superiority of this Z-scheme BCBT heterojunction structure is demonstrated by the remarkable photocatalytic activity. More importantly, the photocatalytic degradation mechanism of the heterojunction is further revealed, which provides guidance for the design of a photocatalyst.

## 2. Experimental

### 2.1. Materials

Melamine, potassium borohydride (KBH_4_), and tetracycline hydrochloride (TC) were purchased from Chinese Medicine Group Chemical Reagent Co., Ltd. (Shanghai, China). Degussa P25 (P25, with 85% anatase and 15% rutile) was purchased from Sigma Aldrich (St. Louis, MO, USA). All chemicals were of analytically pure grade and used without further purification.

### 2.2. Fabrication of Black TiO_2_/B-Doped g-C_3_N_4_ Heterojunction

**Scheme 1:**
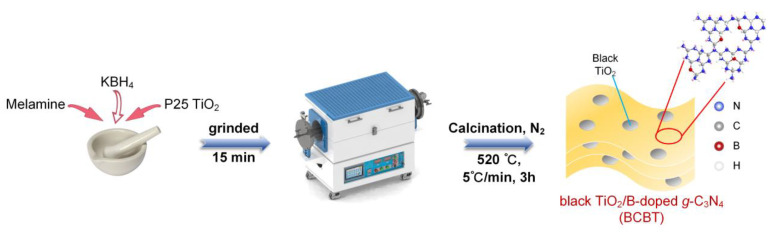
Schematic illustration for synthesis of the black TiO_2_/B-doped g-C_3_N_4_ (BCBT).

Further, 2.5 g Melamine, 2.5 g P25, and 1.0 g KBH_4_ were ground thoroughly for 15 min. Then, the mixture was calcined in a N_2_ flow at 520 °C for 3 h under normal pressure conditions with a constant heating rate of 5 °C min^−1^. The obtained composite was washed with deionized water and ethanol three times, and then dried in an oven at 80 °C overnight. Then, the resulting yellow product of BCBT was collected and ground into powder for further use, as detailed in Scheme 1.

### 2.3. Characterizations

The structure and phase of materials were determined using a SmartLAb SE X-ray diffractometer (XRD, Rigaku, Tokyo, Japan) with Cu-Kα radiation source at an operating voltage of 40 kV and an operating current of 180 mA. ESCALAB Xi+ X-ray photoelectron spectrometer (XPS, Thermo Fisher, MA, USA) with Al-Kα radiation as the excitation source was used to examine the elements on the surface of the samples. On a Regulus 8220 scanning electron microscope (SEM, Hitachi, Tokyo, Japan) and a JEM-2100 transmission electron microscope (TEM, JEOL, Tokyo, Japan), the microscopic morphologies of the samples were examined. The UV-vis diffuse reflectance spectrum (DRS) was recorded in the range of 200~800 nm using a UV 2600 UV-vis spectrophotometer (Shimadzu, Tokyo, Japan) using BaSO_4_ as a reference standard. The photoluminescence (PL) spectra of samples were measured using an LS 55 fluorescence spectrometer (Perkin Elmer, MA, USA) with an excitation wavelength of 350 nm.

### 2.4. Photocatalytic Degradation of Organic Pollutants

Using a Xenon arc lamp (PLS-SXE300+, PerfectLight, Beijing, China) with a cut-off filter (λ > 420 nm) and tetracycline hydrochloride (TC) as a contaminant, the photocatalytic degradation characteristics of BCBT were investigated. Then, 20 mg of the photocatalyst was added to 100 mL of TC solution with an initial concentration of 10 mg L^−1^. The solution was stirred for 20 min in the dark. The solution (5 mL) was filtered every 20 min. TC residuals were detected using a UV spectrophotometer. Pure distilled water was served as a reference sample.

### 2.5. Photoelectrochemical Properties

The photocurrent test, electrochemical impedance spectroscopy (EIS), and the Mott–Schottky plots of the samples were performed on a CHI-660E electrochemical workstation (Chenhua, Shanghai, China). To initiate the photoelectrochemical tests, a Xenon arc lamp (300 W, Beijing Aulight) with a cut-off filter (λ > 420 nm) was used as the light source. We started by dissolving 20 mg of material in ethanol. With an art airbrush, the dispersion was then uniformly sprayed on an FTO glass. Finally, the BCBT-coated FTO glass was calcined at 350 °C for 2 h in a N_2_ environment. The three-electrode electrochemical station included an aqueous Na_2_SO_4_ solution as the electrolyte, a platinum plate as the counter electrode, FTO glass as the photoanode, and Ag/AgCl as the reference electrode. To de-aerate the solution, the electrolyte was purged with N_2_ gas before use.

## 3. Results and Discussion

**Figure 1:**
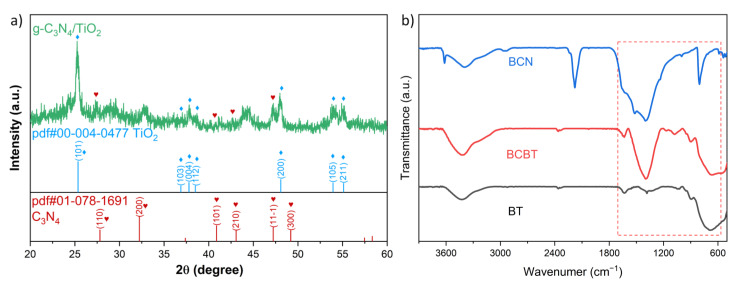
The XRD patterns of BCBT photocatalysts (TiO_2_ in blue, C_3_N_4_ in red) (**a**) and FT-IR spectra of all prepared photocatalysts (**b**).

As shown in Figure 1a, the strong (200) peak at 32.2° and the (100) peak at 15.9° for the BCN (B-doped C_3_N_4_), respectively, belonged to the inter-layer and in-plane crystal facets of g-C_3_N_4_ (ICDD 01-078-1691). The diffraction patterns of the as-prepared b-TiO_2_ were well matched with that of the anatase TiO_2_ (ICDD 00-004-0477), showing that there was no impurity phase introduced after reduction by NaBH_4_. All of the diffraction peaks for the BCBT were identical to those for g-C_3_N_4_ and b-TiO_2_. These results indicate that there are no additional impurity peaks, proving that the B-doped g-C_3_N_4_/black-TiO_2_ (BCBT) composite samples were successfully synthesized via the one-pot process. The FT-IR spectra of the BT (black-TiO_2_), BCN, and BCBT further validated the existence of BCN and BT (Figure 1b). The typical peaks of g-C_3_N_4_ can be observed in BCBT at about 3200 cm^−1^ (C-H) and 1250–1650 cm^−1^ (C-N), which are consistent with the BCN [26]. Additionally, the characteristic peak of Ti-O is found at 500–1000 cm^−1^ [14]. All these findings demonstrate the presence of BCN and BT in the BCBT.

**Figure 2:**
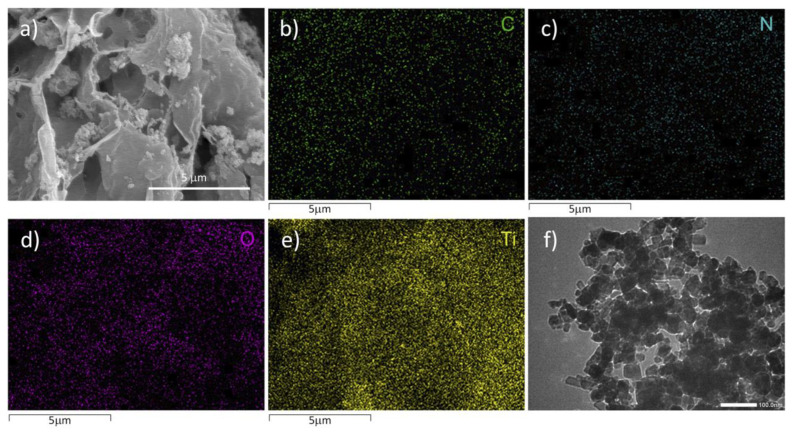
SEM (**a**) and TEM (**f**) images of BCBT; (**b**–**e**) EDX elemental mappings of C, N, O, and Ti.

SEM and TEM were used to characterize the morphology of the obtained samples. In Figure 2a, the thin-layered BCN is associated with the b-TiO_2_ microspheres, which also demonstrates that b-TiO_2_ exhibits microspheres with sizes of about 50 nm. Figure 2a shows that the thin-layered BCN is deposited on the surface of b-TiO_2_ among the BCBT. EDS analysis confirms the existence of Ti, O, B, and C (Figure 2b–e). The TEM image of the BCBT in Figure 2f, which depicts the BCN nanoflakes loaded onto the surfaces of the b-TiO_2_ nanoparticles, further demonstrates this point and is in line with the findings of the aforementioned SEM studies. These findings demonstrate that b-TiO_2_ was successfully attached to the BCN surfaces.

**Figure 3:**
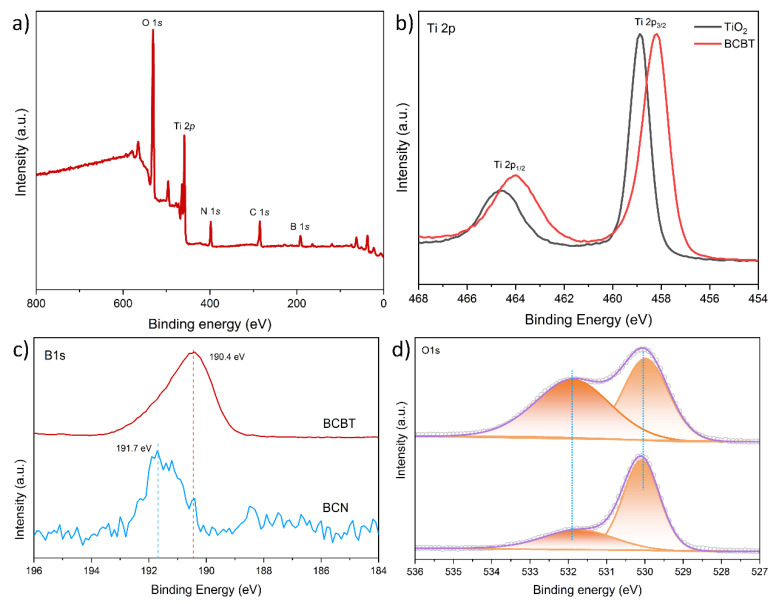
The XPS spectra of BT, BCB, and BCBT: (**a**) survey spectrum of BCT; (**b**) Ti 2p and (**d**) O 1 s of BT and BCBT; and (**c**) B 1 s spectra of BCN and BCBT.

We examined the change of surface chemical bonding of BCBT induced by NaBH_4_ treatment with XPS. The XPS survey spectrum reveals the presence of Ti, B, C, N, and O elements (Figure 3a). A tiny change for Ti 2p can be observed in Figure 3b. This may imply that oxygen-bound electrons bound to titanium and oxygen ions turn in oxygen vacancies, which serve as electron traps [27]. Figure 3c displays the high-resolution B 1s peaks of BCN and BCBT. The BCN peak at 191.7 eV represents the typical B-N bond [28,29]. The BCBT peak at 190.4 eV, with a lower binding energy than BCN, shows that some boron atoms are less electropositive than BCN. These demonstrate the efficient charge transfer in the BCBT between b-TiO_2_ and BCN [30]. A peak near 532.5 eV in the O 1 s (Figure 3d) can be ascribed to adsorbed water, which is consistent with a robust interaction between O vacancy sites and water vapor. This peak area clearly grew during the NaBH_4_ reduction process, which is consistent with the electron transfer to the nearby oxygen vacancies, as shown in the Ti 2p spectrum [31,32,33,34].

**Figure 4:**
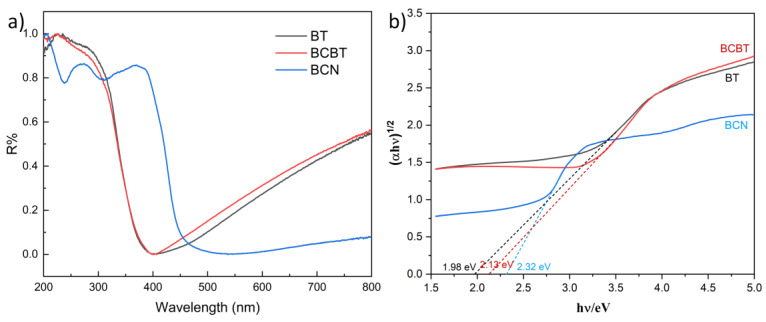
(**a**) UV-vis diffuse reflectance spectra of the as-prepared BT, BCN, and BCBT. (**b**) Relationship of (ahν) ^1/2^ vs. E (ev).

The light absorption ability is one of the crucial factors in determining photocatalytic performance. The light absorption properties of the as-prepared samples were characterized by the UV-vis diffuse reflectance spectra (UV-vis DRS). The absorption edges of BT and BCN, as seen in Figure 4a, are at wavelengths of around 400 and 460 nm, respectively, while the two photocatalysts all broaden the range of visible light absorption following NaBH_4_ reduction. One-pot solid synthesis further enhances the light-harvesting abilities of BCBT, which is attributed to the effective charge transfer between the BCN nanoflakes and BT nanoparticles. An additional broad absorption peak with a wavelength of roughly 400~800 nm is observed in the BCBT hybridized photocatalyst. The O vacancies and doped B elements both promote the activation of BCBT’s e^−^-h^+^ couples when exposed to visible light, increasing BCBT’s sensitivity to light. Figure 4b displays the Kubelka–Munk conversion curves for BT, BCN, and BCBT. Band gaps for BT, BCN, and BCBT are estimated to be ~1.98 eV, 2.32 eV, and 2.13 eV, respectively. According to these results, the BCBT, which has a narrower intrinsic bandgap than that of the BCN, is more active in regions of visible light. As a result, it explains why the subsequent photocatalytic activity was improved. The substantial absorption in the visible light range of BCBT is caused by the existence of oxygen vacancies and doped B elements [33,35]. Combining the characterization findings, it can be concluded that the addition of O vacancy sites and doped B elements increases the catalyst’s ability to absorb visible light, which is obviously conducive to the photocatalytic performance of defective BCBT.

**Figure 5:**
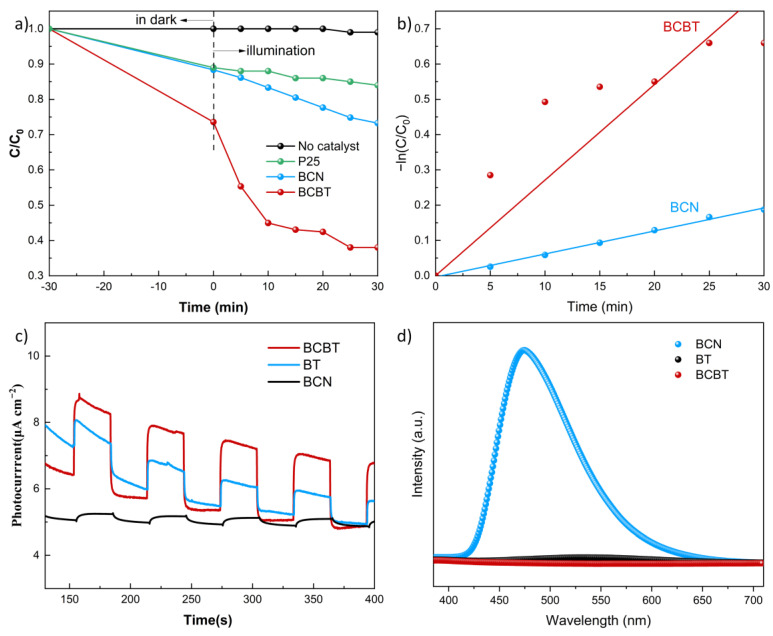
Photocatalytic degradation efficiencies on the degradation of TC under simulated solar light irradiation (**a**), and kinetic linear simulation curves (**b**), photocurrent curves (**c**), and PL spectra (**d**) of BT, BCN, and BCBT, respectively.

The research results of photocatalytic activity of several samples for TC degradation are shown in Figure 5a. The reaction conditions are given in Section 2.2. Figure 5a displays the photocatalytic degradation rate of BCN at 27% after 30 min. The photocatalytic activity of BCBT was improved greatly, and degradation efficiency was up to 65% within 30 min. These may be due to the fact that the addition of OVs increases the light absorption range and creates a BCN/BT heterojunction that encourages photogenerated charge separation (PL and EIS spectra). By developing a kinetic model of the reaction, the kinetic behavior of the photocatalytic degradation reaction may be investigated further below.

−d*C*/d*t* = *kC*/(1 + *kC*) (1)

when *kC*≪1, it can be simplified to pseudo-first-order dynamics.

−d*C*/d*t* = k*C* (2)

where k stands for the pseudo-first-order kinetic constant. The kinetic constants for each molecule are shown in Figure 5b. The simplified pseudo-first-order kinetic formula of L-H demonstrates a remarkable linear relationship between the residual concentration of TC in various samples. The first-order kinetic constants for the fitted kinetic curves of BCN and BCBT in Figure 5b are ~0.0065 and 0.0271 min^−1^, respectively. The kinetic constants of BCBT are 4.17 times higher than that of BCN, indicating that BCBT’s photocatalytic activity greatly increased. Consequently, the BCBT photocatalyst has potential use in wastewater treatment due to its high efficiency, stability, and applicability of antibiotic photodegradation. Additionally, several photocatalysts for the photodegradation of TC published recently are presented in Table 1 and contrasted with the results in this work. The BCBT produced in this work showed superior photodegradation activity with a shorter reaction time when exposed to visible light irradiation when compared to other photocatalysts. This further demonstrates the capability of B-doped g-C_3_N_4_/black-TiO_2_ heterojunction photocatalysts for the photocatalytic degradation of TC.

Transient photocurrent responses, which can be utilized to assess charge-transfer properties and photocatalyst stability, were studied using chronoamperometry. As shown in Figure 5c, BCBT has a higher photocurrent density and electron-hole separation efficiency than BT and BCN due to the presence of O vacancy and the heterojunction formation. Additionally, all composite photocurrent responses for both samples are continuous, demonstrating high stability. Figure 5d depicts the PL spectra of BT, BCN, and BCBT. The results reveal that BCBT has the lowest PL response when compared to the other samples, demonstrating that the photogenerated electron-hole pairs efficiently separate after one-pot solid reduction. Charge separation and transfer can be effectively enhanced by decreasing the recombination rate of the photogenerated carriers, directly boosting photocatalytic performance. Electrochemical impedance spectroscopy (EIS) was also used to analyze the migration of the charge carriers. Evaluating the kinetics at the interface requires a knowledge of the as-synthesis electron transfer resistance, which has been expressed as the diameter of the Nyquist circles. The BCBT sample has the median Nyquist circle diameter in contrast to the BT and BCN samples, as shown in Figure S1. Clearly, combining BCN with BT-rich O vacancies promotes the separation of photogenerated charge carriers.

**Figure 6:**
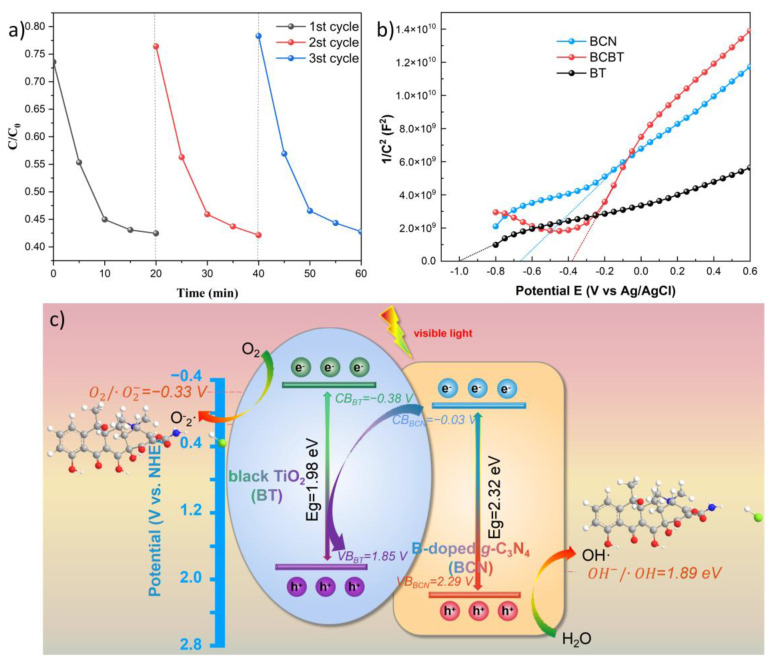
Recycling experiments (**a**), Mott–Schottky plots (**b**) of BCBT, and the presented mechanism (**c**) for photocatalytic TC elimination by BCBT photocatalyst.

Figure 6a shows the degradation and recovery rates after three cycles of using the BCBT photocatalyst. After three cycles, the 30 min photocatalytic degradation efficiency and recovery rate are still 58.2%, which implies that the sample has high stability, implying the potential applications in fields of environment.

The Mott–Schottky (MS) plots for BT, BCN, and BCBT demonstrated that they were typical n-type semiconductors with relatively positive slopes, as shown in Figure 6b. Calculated from x-intercepts of the linear region, the flat-band potentials of BT, BCN, and BCBT were shown to be −1.01 V, 0.66 V, and 0.38 V vs. SCE. As a result, BT and BCN had conduction band potentials (ECB) of −0.38 V and −0.03 V vs. NHE, respectively. Comparing the ECB of BCBT composite to those of BT and BCN, it appears that there was a significant positive movement. The conduction band potential was believed to have shifted positively as a result of the electrical interactions between BT and BCN, leading to a low conduction band position and a higher observable absorption power for the BCBT composite.

The proposed photocatalytic mechanism over the BCBT photocatalyst is shown in Figure 6c. Both the BCN and BT produced photoinduced carriers when exposed to visible light. The photoinduced electrons were then transported from the CB of the B-doped g-C_3_N_4_ to the VB of the black TiO_2_ to create Z-scheme photocatalysts [42,43,44]. In addition, the photogenerated electrons produced by the BT reduced oxygen to form O_2_^−^ (E_0_ (O_2_/O_2_^·−^) = −0.33 eV) [45]. The photogenerated holes produced by the VB of BCN were sufficiently positive to cause the oxidation of OH^−^ to OH (E_0_(OH^−^/·OH = +1.89 eV) [46]. Then, the TC interacted with RSs (reactive species: O^2–^, ·OH, and h^+^) to promote the degradation process. In addition, the absorption of visible light increases when in situ black TiO_2_ is combined with B-doped g-C_3_N_4_. The Ovs level and the introduction of B components considerably increase the BCBT photocatalysts’ ability to absorb light, giving them exceptional photo-absorption properties.

## 4. Conclusions

In conclusion, the successful synthesis of the Z-scheme black TiO_2_/B-doped g-C_3_N_4_ heterojunction photocatalyst and evaluation of the photocatalytic processes were accomplished. Black TiO_2_/B-doped g-C_3_N_4_ had a higher photocatalytic activity than black TiO_2_ and B-doped g-C_3_N_4_. The TC removal ratio for BCBT reached up to 65% within 30 min, which was much higher than that for pure BT and BCN. The abundance of OVs and B-doped elements in BCBT was largely responsible for its outstanding photocatalytic activity. These led to effective photogenerated carrier separation and enough visible light absorption, which improved BCBT’s photocatalytic efficiency.

## Figures and Tables

**Table 1:** The comparison of photocatalytic degradation activities of different photocatalysts for TC.

Photocatalyst	Light Source	Dosage of Catalyst (g L^−1^)	TC Concentration (mg L^−1^)	Reaction Time (min)	Rate (min^−1^)	Reference (year)
C nanodots/WO_3_	150 W XL (λ > 420 nm)	0.5	20	150	0.0067	[36] (2017)
Ag/a-NiMoO_4_ nanorods	150 W XL (λ > 400 nm)	1.429	20	180	0.0093	[37] (2019)
C-TiO_2_ nanocomposites	visible-light	0.2	10	160	0.0126	[38] (2019)
BiOCl/TiO_2_ C nanofibers	300 W XL (λ > 420 nm)	0.5	20	180	0.0085	[39] (2020)
ZnSnO_3_/g-C_3_N_4_	300 W XL (λ > 420 nm)	0.25	10	120	0.0131	[40] (2020)
C-doped 0.5-UNST	300 W XL (λ > 420 nm)	0.5	20	120	0.0134	[41] (2021)
BCBT	300 W XL (λ > 420 nm)	0.2	10	30 (60%)	0.0271	This work

## Data Availability

The raw/processed data required to reproduce these findings cannot be shared at this time due to technical or time limitations.

## Supplementary Materials

The following supporting information can be downloaded at: https://www.mdpi.com/article/10.3390/nano13030518/s1, Figure S1: EIS plot of BT, BCN and BCBT, respectively.
