# Supplementary material for: B-Doped g-C3N4/Black TiO2 Z-Scheme Nanocomposites for Enhanced Visible-Light-Driven Photocatalytic Performance
Source: Nanomaterials (Basel). 2023 Jan 28;13(3):518. doi: 10.3390/nano13030518 (PMC9920186; doi:10.3390/nano13030518)
Supplement: Supplementary file 1 [file nanomaterials-13-00518-s001.zip › nanomaterials-2163007-supplementary.pdf]

Supplementary Material

B-Doped g-C_3N_4/Black TiO_2 Z-Scheme Nanocomposites for Enhanced Visible-Light-Driven Photocatalytic Performance

Yuwei Wang *, Kelin Xu, Liquan Fan *, Yongwang Jiang, Ying Yue and Hongge Jia

Heilongjiang Provincial Key Laboratory of Polymeric Composite Materials, College of Materials Science and Engineering, Qiqihar University, Qiqihar 161006, China; xushaoyi1998@163.com (K.X.); jiangyongwang6245@163.com (Y.J.); 18814663520@163.com (Y.Y.); jiahongge@qqhru.edu.cn (H.J.)

* Correspondence: ywwang@qqhru.edu.cn (Y.W.); 02275@qqhru.edu.cn (L.F.)

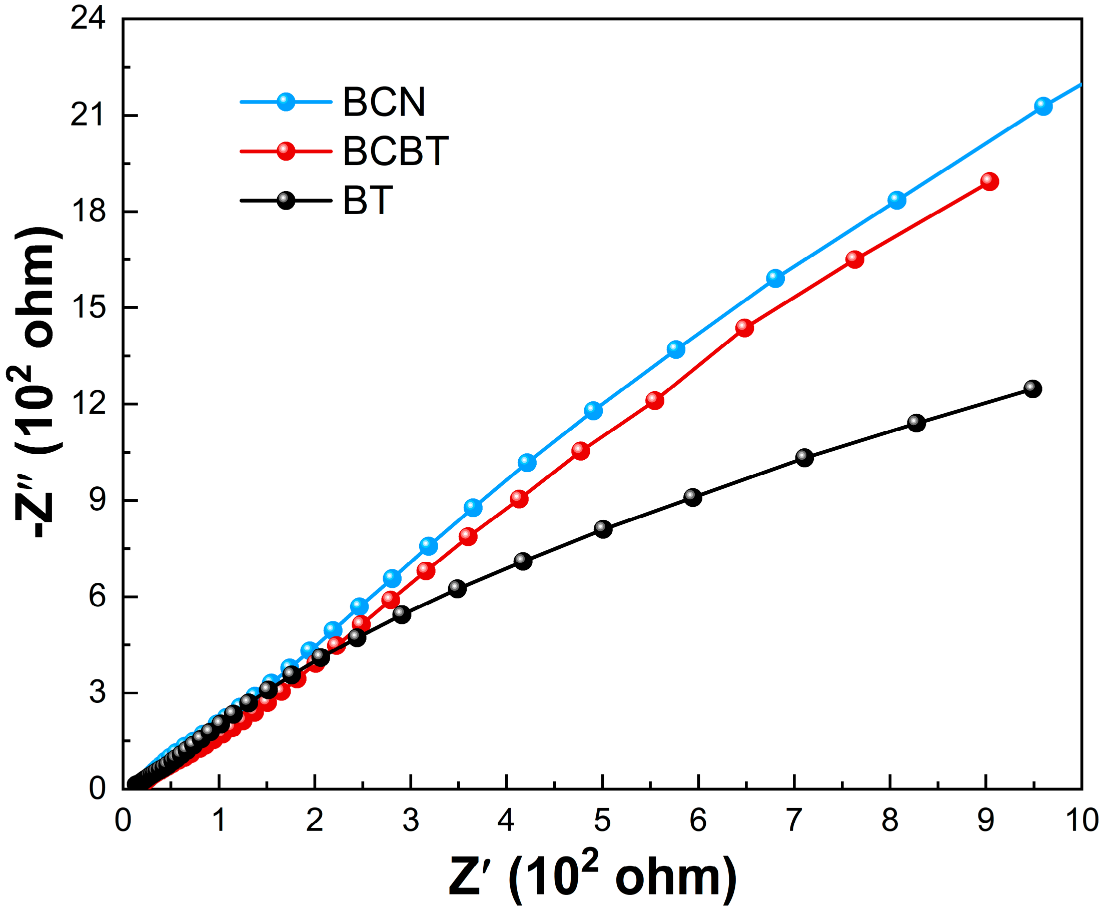

Figure S1. EIS plot of BT, BCN and BCBT, respectively.
